# The Role of Interhemispheric Interactions in Cortical Plasticity

**DOI:** 10.3389/fnins.2021.631328

**Published:** 2021-07-09

**Authors:** Jan Antoni Jablonka, Robert Binkowski, Marcin Kazmierczak, Maria Sadowska, Władysław Sredniawa, Aleksandra Szlachcic, Paulina Urban

**Affiliations:** ^1^Faculty of Biology, University of Warsaw, Warsaw, Poland; ^2^Dominick P. Purpura Department of Neuroscience, Albert Einstein College of Medicine, New York, NY, United States; ^3^Nencki Institute of Experimental Biology of Polish Academy of Sciences, Warsaw, Poland; ^4^College of Inter-Faculty Individual Studies in Mathematics and Natural Sciences, University of Warsaw, Warsaw, Poland

**Keywords:** cortical plasticity, barrel field, 2DG, 2-deoxy-D-glucose, interhemispheric

## Abstract

Despite the fact that there is a growing awareness to the callosal connections between hemispheres the two hemispheres of the brain are commonly treated as independent structures when peripheral or cortical manipulations are applied to one of them. The contralateral hemisphere is often used as a within-animal control of plastic changes induced onto the other side of the brain. This ensures uniform conditions for producing experimental and control data, but it may overlook possible interhemispheric interactions. In this paper we provide, for the first time, direct proof that cortical, experience-dependent plasticity is not a unilateral, independent process. We mapped metabolic brain activity in rats with 2-[^14^C] deoxyglucose (2DG) following experience-dependent plasticity induction after a month of unilateral (left), partial whiskers deprivation (only row B was left). This resulted in ∼45% widening of the cortical sensory representation of the spared whiskers in the right, contralateral barrel field (BF). We show that the width of 2DG visualized representation is less than 20% when only contralateral stimulation of the spared row of whiskers is applied in immobilized animals. This means that cortical map remodeling, which is induced by experience-dependent plasticity mechanisms, depends partially on the contralateral hemisphere. The response, which is observed by 2DG brain mapping in the partially deprived BF after standard synchronous bilateral whiskers stimulation, is therefore the outcome of at least two separately activated plasticity mechanisms. A focus on the integrated nature of cortical plasticity, which is the outcome of the emergent interactions between deprived and non-deprived areas in both hemispheres may have important implications for learning and rehabilitation. There is also a clear implication that there is nothing like “control hemisphere” since any plastic changes in one hemisphere have to have influence on functioning of the opposite one.

## Introduction

**FIGURE 1:**
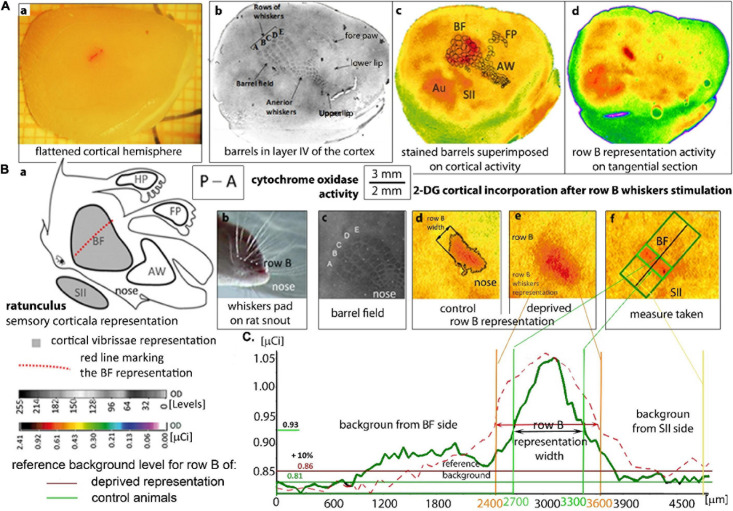
Localization and measurement of the row B cortical representation: **(A)** A flatted hemisphere and tangential sections of the cortex after 2DG brain activity mapping. **(A,a)** Isolated hemisphere with a marked line perpendicular to the representation in row B. Localization of the rats’ cortical whiskers somatosensory representations on a tangential section of layer IV **(A,b–d)** stained by cytochrome oxidase activity **(A,b)** and 2DG-incorporation on autoradiograms visualizing the brain activity **(A,c,d)**. **(A,c)** Superimposed barrels from the histological staining to the suitable autoradiogram image which is shown in pseudocolors. **(A,d)**: the autoradiogram presenting stimulated row B activity. (AW) anterior whiskers representation; (FP) front paw representation; (SII) secondary somatosensory cortex, (Au) auditory cortex which is always activated by the white noise. The left whiskers stimulation in intact rats activates, the right BF and SII **(A,c)** in the contralateral hemisphere and do not activate AW, hind paw and FP cortical representations **(A,c,d)**. **(B)** The cortical vibrissal representation in SI and SII. **(B,a)** “Rattunculus” with areas of increased activation during all whiskers stimulations marked in gray. **(B,b)** Photograph of the whiskers pad on the rat snout showing row B. **(B,c)** Magnification of the barrel field on the cortical tangential section stained for cytochrome oxidase activity. **(B,d,e)** A magnification of row B cortical representation on digitized autoradiograms of tangential sections in undeprived **(B,d)** and deprived **(B,e)** animals. **(B,f)** Row B representation with the marked area of the width measured with the rotatable box. **(C)** Comparison of the OD graphs showing 2DG incorporation from the animals with non-deprived (green line) and deprived BF (dashed red line). OD analysis by the MCID program, where the area exceeding the background over 10% were treated as the activated representation of row B. The mean value of OD from the neighborhood of the BF (right side of the graph, from the SII side of the stimulated row B) was treated as a reference area. The subscribed “background from the BF side” was altered by the whiskers stimulation. OD—optical density in arbitrary units with a direct set of 255 gray scale levels or calibrated to μCi radiation units of [^14^ C]-2DG; P–A—posterior to anterior.

Rodents whiskers’ representation in the somatosensory cortex is widely used as a model of cortical plasticity due to its highly somatotopic organization. Each whisker has its sensory representation in the cortical area of the barrel field (BF) in the contralateral hemisphere (Figure 1A). Each barrel is a representation of one whisker. Barrels are easily visualized in layer IV and are constructed by a dense composition of cells around the middle part of columnar representation of the whisker. The barrels are arranged in the cortex like the whiskers on the snout (Fox and Woolsey, 2008), and this makes them a good model for cortical map remodeling. The whisker sensory information passes through the ventroposteromedial (VPM) and the medial division of the posterior nucleus (POm) in the contralateral thalamus, reaching, respectively, the barrel center and the septas between barrels (Kim and Ebner, 1999). These two distinct thalamocortical systems are supposed to be separate channels for processing sensory information, since signals from the two systems are combined in SII (Alloway, 2008; Feldmeyer, 2012). The sensory information from the ipsi- and contralateral cortices is supposed to be fused for the very first time when transferred calossally between SI areas (Fabri et al., 2005) and SII (Carvell and Simons, 1987). The cortical sensory information input from the whiskers in rodents is supposed to be completely crossed (White and DeAmicis, 1977; Simons and Woolsey, 1979; Olavarria et al., 1984; Koralek et al., 1990; Cauller et al., 1998; Renier et al., 2017) and at the same time requires representations in both hemispheres for the animal to perform bilateral tactile discrimination (Shuler et al., 2001, unilateral tasks are not supposed to engage the ipsilateral SI; Hutson and Masterton, 1986).

Like other primary representations, the cortical columns in the BF are involved in the first step of cortical information processing. The sensory input arrives to layer IV in the BF, goes through circuits of columnar loops of cellular connections and is combined with the thalamocortical and cortico-cortical loops that modify the contextual response (Alloway, 2008). Prolonged changes in the sensory input from the whisker pad can be observed as connectivity remodeling in the well-defined structure of BF. It was shown (in rats) that a month of unilateral, partial whiskers deprivation results in the widening of the spared whiskers representation at the expense of surrounding area of the deprived whiskers representations (Kossut et al., 1988; Diamond et al., 1993; Fox, 1994). The widening can be visualized by [^14^C]-2-deoxyglucose (2DG) mapping of the metabolic correlates of brain activity (McCasland and Woolsey, 1988).

The enlargement of one representation in response to surrounding whiskers deprivation that is visualized by 2DG was already shown by Dietrich et al., 1985. Since that time it has been widely used in experiments exploring plasticity mechanisms in the healthy and injured cortex. However, the difference in response to uni- vs. bilateral stimulation was hardly considered and studied. Simultaneous bilateral stimulation of corresponding rows of whiskers resulted in a similar area of 2DG incorporation in layer IV and more superficial layers in both hemispheres (Siucinska and Kossut, 2004). A unilateral stimulation led to 2DG incorporation only in the contralateral hemisphere, a result that is in accordance with the theory of full crossing of this sensory pathways (Debowska et al., 2011). However, the two hemispheres were usually treated as independent even in experiments involving unilateral, peripheral and/or cortical manipulations (Fusco et al., 2003; Sun et al., 2003; Jablonka et al., 2007, 2012; Chung et al., 2013). Bilateral whiskers’ stimulation was often used to visualize the cortical plasticity changes in one hemisphere and was compared to the opposite hemisphere, which was treated as a control (Jablonka et al., 2007, 2012; Kaliszewska et al., 2012). In this experimental model, the unilateral partial whiskers deprivation induces plastic changes in the contralateral hemisphere, increasing it by almost 50% when visualized by 2DG incorporation.

It has been shown that the transfer of the effect of learning between BFs in both hemispheres is topographically arranged and is more closely related to strongly connected homotopic whiskers (Harris and Diamond, 2000). Although interhemispheric cortical interactions were reported (Iwamura, 2000; Innocenti, 2009; Ragert et al., 2011) and the inhibitory and excitatory influences of the interactions were shown for a few modalities (Bloom and Hynd, 2005), the possibility that opposite homotopic regions may participate in the remodeling of any representations was rarely considered. Wiest et al. (2005) suggested a revision to the concept of the highly segregated hemispheric processing in BFs and presented data supporting the conjecture that callosal connections constitutively mediate the activity of BF. Glazewski et al. (2007) demonstrated that ipsilateral whiskers can constrain the experience-dependent enlargement of the cortical representation during chronic, unilateral, partial whiskers deprivation. Since it was shown that the information processed in SII may interfere with the contralateral plasticity mechanisms in the BF (Debowska et al., 2011), we hypothesized that interhemispheric interactions may be engaged in the cortical circuits’ remodeling following experience-dependent plasticity in SI as well. We therefore compared metabolic activity during bilateral and unilateral whiskers stimulation for the areas newly engaged in information processing following 1 month of deprivation. We also checked if the changes observed in one hemisphere are somehow reflected in the contralateral homotopic area the undeprived one. To ensure whether the changes observed in one hemisphere are somehow reflected in the contralateral homotopic area the undeprived one row B unilateral whiskers stimulation was performed.

Specifically, we wanted to see if the response to an acute unilateral whiskers stimulation after chronic experience-dependent plasticity induction results in the same pattern of plastic rearrangement of the spared whiskers cortical representations as that resulting from bilateral stimulation.

## Materials and Methods

### Animals

Male Wistar rats weighing about 300 g from the Bialystok colony were used. Animals were housed separately in plastic cages in a 12 h light-dark cycle at approximately 20°C, and had a free access to food and water. There were six groups of rats: three experimental groups with whiskers deprivation (D) of which two groups received unilateral whiskers stimulation (*n* = 6) and one group received bilateral stimulation (*n* = 8) during brain activity mapping; there were also three *naïve* control groups (*n* = 6) where individuals did not receive sensory deprivation (C) but received the same type of whiskers stimulation as the experimental groups. Animals were handled and habituated to the restraining procedure for 1 week before deprivation. All the procedures were accepted by the First Regional Ethical Commission in Warsaw (270/2012) and were in accordance with the European Communities Directive (86/609/EEC).

### Unilateral Partial Whiskers Deprivation

Sensory deprivation was performed by trimming all the left whiskers except for row B. The trimming was repeated every second day for 1 month.

### Brain Mapping

After 4 weeks of vibrissae deprivation, 2DG functional brain mapping was performed (Figures 1Ac,d,Bd–f,C; Kennedy et al., 1975). Animals were put in a restrainer and the whiskers of the undeprived right side were cut close to the skin apart from row B. [^14^C] – 2DG (7 μCi/100 g b.m., American Radiolabeled Chemical, spec. act. 55 mCi/mmol; St. Louis, MO, United States) was injected intramuscularly. During brain mapping the whiskers of the both rows B were stroked manually uni- or bilaterally in experimental and control groups, in rostro-caudal direction with frequency of 2 Hz. After 30 min of stimulation the animal was deeply anesthetized by intraperitoneal injection of Morbital (0.1 ml/100 g).

### Tissue Histological Preparation

Rats were killed by i.p., Morbital injection (0.1 ml/100 g; Biovet, Pulawy, Poland) and intracardially perfused with 4% paraformaldehyde (Sigma-Aldrich; St. Louis, MO, United States). Brains were removed and cortices of separated hemispheres were flattened between glass slides to 3 mm, snap frozen in heptane at −70°C and stored at −80°C. Hemispheres were cut tangentially (Figure 1Aa) on a cryostat at −16°C into 20 μm tangential sections, which were collected alternately on specimen slides and cover slips. The sections, which were on cover slips were immediately dried and exposed on an X-ray film (MIN-R 2000; Kodak) for 3 weeks with a set of [^14^C] standards (American Radiolabeled Chemicals; St. Louis, MO, United States). The remaining sections were stained for cytochrome oxidase (CO) activity to identify the BF (Figures 1Ab,Bc), and the BF marked perpendicularly to row B, with DiI (Figure 1Aa).

The histological staining images of tangential sections with the barrels present for HP, BF and anterior whiskers were superimposed on corresponding autoradiogram images which mark the regions of the sensory representations.

### Data Analysis and Statistics

The autoradiograms were analyzed by a computer image analysis program (MCID; InterFocus Ltd., Cambridge, United Kingdom). The optical density (OD) was measured by the program with 8 bits accuracy with 255 gray scale levels and OD and distance calibration were performed. The software allowed us to display an image of the section stained for CO activity next to the section from which the autoradiogram was obtained and to superimpose these images on each other so that the position of the barrel field, hind limb, forepaw, anterior whiskers and the relation of the other areas can be precisely defined.

After establishing the OD of the autoradiograms within the range of [^14^C] standards and the increase of OD in a linear standard manner, autoradiograms were compared with stained brain sections (Figure 1Ab,c). Assuming that the OD in autoradiograms is proportional to the concentration of the [^14^C] isotope (that in turn reflects local glucose consumption; Sokoloff et al., 1977), we used the μCi values as a direct measure of cortical activity. We converted the OD values from digitized images into μCi based on the ODs of the [^14^C] standards that were co-exposed with the cortex sections. The software measured OD values in labeled regions. The geometric properties of the marked areas were measured automatically by the software or with the software tools. The width of the labeled cortical representation of row B whiskers was measured in layer IV. The pixels with 2-DG-uptake intensity that were consistently above 15% of the mean surrounding cortex incorporation were considered as a labeled representation (Chmielowska et al., 1986). The width was taken by rotatable box tool crossing perpendicularly to the middle of the delineated region. The results were averaged for all sections from layer IV, II/III, and V/VI of one hemisphere.

The differences between the groups were examined using a multi-factor ANOVA and a *post hoc* Tukey test (for unequal N). The data were also compared by using bootstrap analysis in 1000 iterations with replacement (Efron, 1979). The significance of the differences between hemispheres of bilaterally stimulated rats was calculated with a two-tailed paired Student’s *T*-test.

## Results

### 2DG Cortical Activity Mapping

Uni- and bilateral whiskers stimulations lead to a different pattern of experience-dependent plasticity effects. In these two cases the visualization of the experience dependent plasticity by 2DG incorporation differed significantly (*p* ≤ 0.05). Both types of stimulation generated a band of the representation of sensory row B in the BF that is contralateral to the stimulated rows and more laterally in the neighboring SII (Figures 1Ac,d). However, there were differences in rows B representations visualized in uni- and bilaterally stimulated rats in the deprived animals.

### Row B Representation Width

**FIGURE 2:**
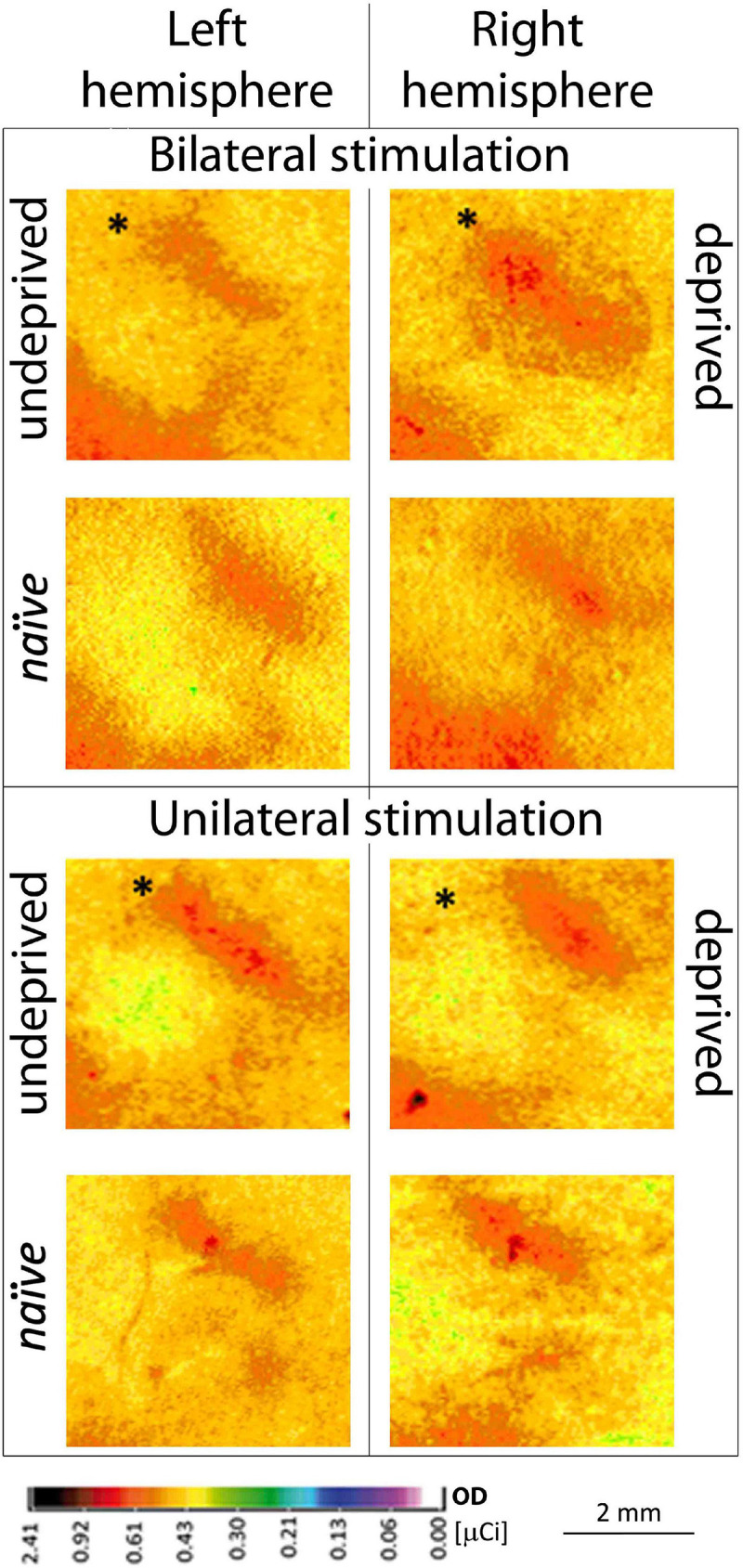
Contralateral 2DG incorporation of row B representations in the two hemispheres during uni- vs. bilateral whiskers stimulation in deprived experimental groups. Individuals with unilateral, partial whiskers cut and bilateral stimulation (*n* = 8) or individuals with unilateral stimulation (*n* = 6) of whiskers’ row B during brain activity mapping are shown; bottom row: naïve animal; ^∗^Mean ± SD; ^∗^*P* < 0.05.

**FIGURE 3:**
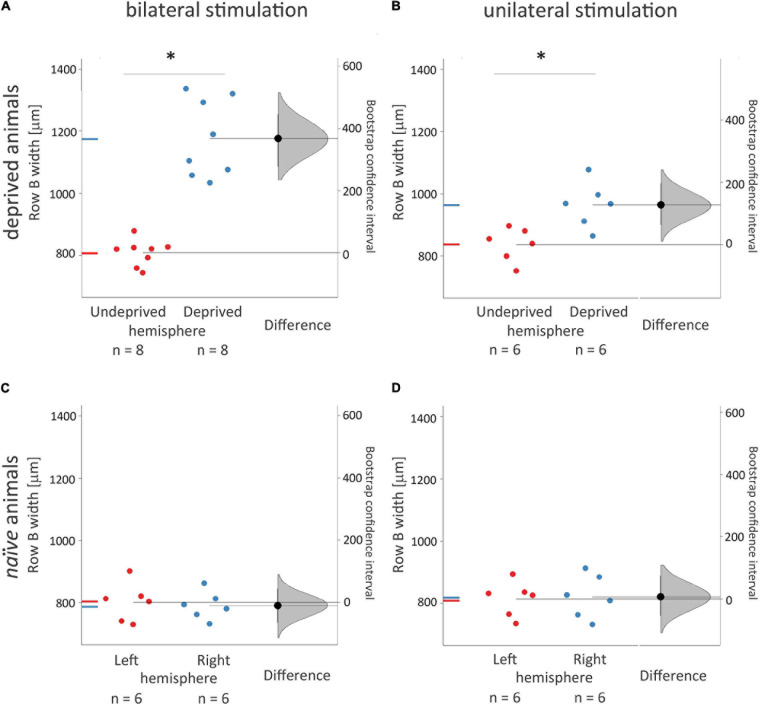
Width of 2DG incorporation in rows B representations in the contralateral, deprived, and non-deprived hemispheres and its dependence on uni- vs. bilateral whiskers stimulation. Experimental groups were deprived and bilaterally (**A**. *n* = 8) or unilaterally (**B**. *n* = 6) stimulated during brain activity mapping; *naïve* rats (**C**.,**D.**) received just whiskers stimulation during brain activity mapping. ANOVA with *post hoc* Tuckey test and bootstrap analysis were used. The right curve shows an estimation plot emphasizing the effect size – the difference between the group means of the left and right hemispheres. The 0 point of the “difference axis” (on the right) is based on the mean of the reference group (the *naive* control group, presented below). The filled triangles show the difference between left and right hemispheres, with 95% confidence intervals. The curves illustrate the range of expected sampling error in estimating the mean difference. *Mean ± SD; *P < 0.05.

Autoradiograms analysis showed differences in rows B representations width between groups (Figures 2, 3; *F*_(__4_,_24__)_ = 17, *p* < 0.0001). In the control *naïve* animals rows B representations’ width in both hemispheres after uni- and bilateral stimulations (Supplementary Table 1) were similar to that in undeprived hemisphere of deprived animals, i.e., around 800 μm (Figure 3; 796 ± 47 vs. 833 ± 71 μm).

The stimulated deprived, left row B representation width in the right hemisphere after unilateral the left whiskers stimulation was 22% smaller than after the bilateral one (Figure 2; 964 ± 73 vs. 1176 ± 125 μm, *p* = 0.02). In unilaterally stimulated rats, the width of the cortical representation of the spared row B in the right hemisphere contralateral to the deprived whiskers pad was 15% wider than that seen in the ipsilateral to the deprived whiskers, the left hemisphere after contralateral (the right) row B whiskers’ stimulation (Figures 2, 3.; 835 ± 65 vs. 964 ± 73 μm, *p* = 0.03), while in bilaterally stimulated rats it was 45% wider (Figures 2, 3; 808 ± 42 vs. 1176 ± 125 μm, *p* < 0.001; similar to the magnitude of the response previously reported by Jablonka et al., 2007, 2012). The relations were similar for all the layers (Supplementary Table 1 and Supplementary Figure 1). To ensure whether the changes in one hemisphere are somehow reflected in the contralateral homotopic area unilateral whiskers stimulation of the undeprived row B was performed and results compared to those of *naïve* animals. There were no significant differences.

### Ipsilateral Response

**FIGURE 4:**
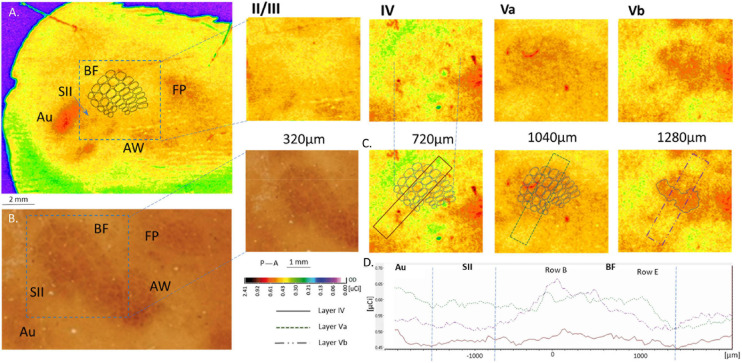
Cortical 2DG incorporation following the ipsilateral row B whiskers stimulation shown in four layers of the barrel field cortex. **(A)** no response is seen in layers II/III and IV (slightly higher incorporation might be observed in deep layer IV). Layer Va shows evident excitation in the entire surface of the BF with stronger activation of row B representation in layer Vb; **(B)** BF in layer IV stained for cytochrome oxidase activity. **(C)** OD measured across the BF in layer IV, Va and Vb. **(D)** 2DG incorporation in the above chosen areas in the tree layers.

When rats were stimulated unilaterally, also ipsilateral activity appeared in the supragranular layers (Figure 4, >1000 μm) of the entire BF in the hemisphere ipsilateral to the whiskers stimulation (mean area 1340 ± 200 μm^2^). Activity was observed in both deprived and undeprived animals and had a uniform 2DG incorporation (1.24 ± 0.03 μCi) just below 1000 μm. In deeper layers activity was smaller and more focused (549 ± 240 mm^2^; *p* < 0.001): it was restricted to the area homotopic to the stimulated row B representation (Figure 4). No statistically significant differences were observed between groups that were unilaterally stimulated. However, the bilateral stimulation gave more focused response in the area restricted to the row’s representation in the supragranular layers (Figure 4; 1.18 ± t0.08 uCi, 229 ± 68 mm^2^, *p* < 0.0001).

## Discussion

Our results show that the cortical areas engaged in experience-dependent plasticity cortical map remodeling are differently activated during uni- and bilateral stimulation, thus suggesting contralateral hemisphere participation in cortical plasticity. We assume that the undeprived hemisphere, which is active all the time, influences the deprived areas of the deprived hemisphere. The result is, as we have shown in this paper, that the neighborhood of the active non-deprived row of whiskers is activated by contralateral homotopic cortical area stimulation in the undeprived hemisphere. It is possible, however, that what we actually observe is a plasticity effects of the inhibitory loop from the contralateral hemisphere, activating the inhibitory interneurons surrounding the only active area in the deprived hemisphere. The control groups showed no such effect. 2DG incorporation, which visualizes the overall effect of inhibitory and excitatory activity as well as the neuronal and astrocyte metabolic response to neuronal activity (Kennedy et al., 1975; Sokoloff et al., 1977), showed deprivation-induced enlargement of the area around the spared + depending on whether unilateral or bilateral whiskers’ stimulation was applied within the area engaged in experience-dependent plasticity.

The 2DG visualized emanation of experience-dependent plasticity cortical map remodeling after partial whiskers deprivation differed following the two treatments: the 2DG incorporation area of cortical representation of the spared whiskers was smaller by 22% after contralateral whiskers stimulation than that manifest after bilateral stimulation, although both were enlarged in comparison to the representations in intact animals (by 45 and 15%, respectively). Since it is assumed that information transferred from the vibrissae to the cortex crosses between hemispheres through the corpus callosum (Smith, 1973; White and DeAmicis, 1977; Simons and Woolsey, 1979; Olavarria et al., 1984; Koralek et al., 1990; Cauller et al., 1998; Renier et al., 2017) any additional response presented after bilateral stimulation (in comparison to unilateral stimulation) must be due to transfer through callosal connections.

The corpus callosum is the biggest connective pathway between the brain hemispheres. In the sensory areas the callosal connections are mainly between layers II/III and layer V (Huang et al., 2013; Decosta-Fortune et al., 2015). Neurons responsible for callosal connections can also innervate other associative areas like SII (Krubitzer and Kaas, 1990), and callosal connections were suggested to transmit both inhibitory and excitatory stimulations (Kobayashi and Pascual-Leone, 2003; Bloom and Hynd, 2005). The conclusion that the corpus callosum is an active structure, constitutively establishing interhemispheric integration (Hellige, 1993; Banich, 1995; Tame et al., 2016) is inescapable.

The diversity of the signals communicated through the corpus callosum may depend on the context in which excitatory and inhibitory interhemispheric interactions occur (Banich, 1995; Cooke and Bear, 2013; Reed et al., 2010, 2011). This means that there are grounds to believe that both the inhibitory and excitatory signals transferred through callosal fibers influence innervated regions in the cortex (Hlushchuk and Hari, 2006). Previous studies demonstrated the direct influence of contralateral homotopic regions on the activity patterns of sensory representations (Meissirel et al., 1991; Clarke and Zaidel, 1994; Iwamura, 2000) and it was shown that sensory information from the whiskers can be modulated by incoming information from the contralateral BF, probably via direct connections through callosal afferenciation of both BFs (Li et al., 2005).

Celikel et al., 2004 demonstrated the immediate changes after deprivation in connectivity of layer IV and II/III, and Li et al. (2005) also showed that the unilateral suppression of BF activity modifies responses to incoming information in the contralateral BF with no impact on spontaneous activity. However, as far as we know, there were as yet no studies (except one, Glazewski et al., 2007) that supported the idea that the constitutive cooperation between the hemispheres may contain cortical remodeling through neuronal plasticity mechanisms in SI. The results presented in this paper further support the idea that the rewiring of the deprived hemisphere has some influence on the contralateral one and that there are on-going re-entrant interactions between the two hemispheres. They also suggests that incoming information processing in the “intact” hemisphere of deprived animals is likely to differ from information processing in *naïve* (intact) animals.

The response of the cortical neurons in ipsilateral layer V was described by Shuler et al. (2001), who showed that ipsilateral input from multiple whiskers increases the probability of spike recording in direct proportion to the amount of whiskers’ stimulation. In the experiments described in this paper, continuous stroking for half an hour of one row of 5 whiskers gave a uniform 2DG incorporation in the entire BF of the ipsilateral hemisphere in layer Va. This illustrates the active connectivity of the transcallosal axons. The transcallosal connections may trigger the induction of background activity, changing the sensitivity of the whole BF to the processing of signals entering layer IV, which becomes integrated with the current activity induced by the stimulation of the ipsilateral whiskers. The pattern of 2-DG incorporation does not, however, provide information regarding the electrophysiological properties of the stimulus-related activity. Nevertheless, analysis of the post-stimulus latency and time histograms for multi-unit activity showed responses in layer V of the principal and neighboring barrels as well as in the surrounding septal regions. Contralateral whisker stimulation resulted in a greater receptive field of the cells in layer V than that of the cells in layer IV (Wright and Fox, 2010).

Our results clearly show that 2DG incorporation in layer Vb and VI was targeted onto the area homotopic to the stimulated contralateral cortical representation of the stroked ipsilateral whiskers’ row B (Figure 3). This result may seem contrary to the cellular recordings reported by Wright and Fox (2010), who showed lower septum/barrel response differentiation in layer Vb than in Va; however, since they focused on contralateral responses while we applied ipsilateral whiskers stimulation, there is no contradiction. Moreover, Wright and Fox (2010) recordings showed a lower range of differences than the 2DG-incorporation detection threshold.

**FIGURE 5:**
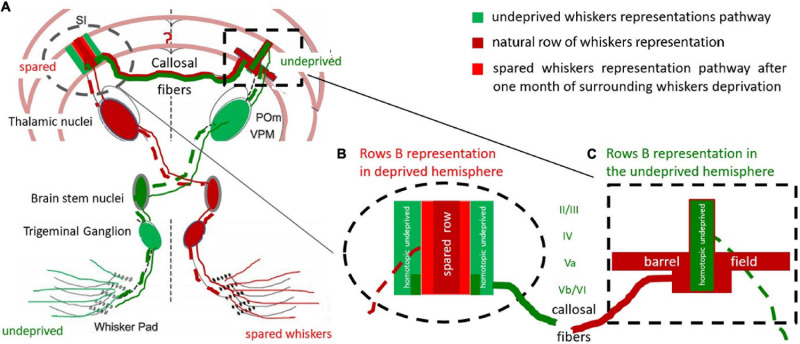
Hypothetical reconstruction of the ExDP in the cortex of BF. **(A)** Whiskers-BF pathway. The cortical row of whiskers’ representations shows the profile of the activation perpendicular to the row during bilateral whiskers stimulation of spared and homotopic contralateral row of whiskers; **(B)** cortical representation of a spared row of whiskers. **(C)** BF with contralateral, homotopic to the spared row of whiskers, row B cortical representation (green), and spread activation in response to ipsilateral spared whiskers stimulation (red); Red: response to unilateral, spared whiskers’ stimulation, from the deprived whisker pad; Dark red: response typical to the stimulation of undeprived row of whiskers (when the whisker pad is intact); Green: undeprived whiskers activity effects in deprived animals during bilateral homotopic rows stimulation (the undeprived and spared one). The homotopic undeprived row B stimulation effect on (the green on Figure 5B) is supposedly the transcallosal inhibition from activated during the deprivation period homotopic to the spared one row of whiskers. dashed line – contralateral whiskers-BF pathway; solid line – ipsilateral pathway; POm – Posteromedial nucleus; VPM – ventroposteromedial nucleus. Adapted from Shuler et al., 2001.

The homotopic characteristic of the transcallosal connections in the subgranular layers of the BF was described by Chovsepian et al. (2017). This characteristic corresponds in our study to the hyperactivity of layer Vb of row B representation, which changed smoothly to encompass the entire BF activation in layer Va (Figures 4, 5). It is also compatible with the time points of responses recorded by Wright and Fox (2010), who showed 0.7 ms earlier activation in layer Vb than in Va. This suggests that incoming transcallosal information was first transmitted to the homotopic area of layer Vb, and was later distributed to the entire BF in the Va layer *via* one or two synapses. Since most sensory areas propagate their callosal connections to layer V (Decosta-Fortune et al., 2015) this could reflect the transfer of incoming information from the stimulated hemisphere to the contralateral one, which influence the propensity of the contralateral homotopic areas to respond to any other incoming activation. A hypothetical reconstruction of this processes in presented in Figure 5.

The experience-dependent plasticity we observed is probably the consequence of the lack of counterbalancing equilibria of the circuits in the deprived hemisphere (Barnes et al., 2015; Iwamura et al., 2001). This may explain why only the areas included in the spared row whiskers’ representation were susceptible to type of the stimulation in deprived animals. The ipsilateral response observed in layer V provides additional support to the suggestion that there is ongoing interhemispheric cooperation during unilateral whiskers stimulation (Figure 5). Although we did not observe any differences in the undeprived hemisphere, SII may participate in the interhemispheric integration of plasticity changes (Debowska et al., 2011). We demonstrated that the effect of experience-dependent plasticity in SI, which is visualized by 2DG might be mainly the result of modified interhemispheric interactions. The profile of the 2DG incorporation shows that layer V participates in the integration of incoming sensory information, although our results cannot prove that the ipsilateral responses were modified by experience-dependent plasticity.

Although the effects of the feedback relations between the hemispheres require additional exploration, our results clearly show that the responses of the stimulated ipsilateral whiskers participate in experience-dependent rearrangement of cortical map representations most probably *via* the contralateral hemisphere inhibitory feedback loops (see Palmer et al., 2013; Kokinovic and Medini, 2018). Part of the enlargement would be a result of the direct enlargement of the area processing the incoming stimulation – the area which belongs to the cortical representation of the spared whiskers – and the rest could be due the inhibitory activities around it (Sachdev et al., 2012), which result from the feadback of ipsilateral homotopic to the deprived cortex activity (Figure 5). It seems that both this parts undergow rearangemant after partial whiskers deprivation.

In the visual cortex monocular deprivation results in increase of GABA_*B*_ inhibition (Kokinovic and Medini, 2018) and since the inhibition is greater and imbalanced only in binocular cortical areas (Iurilli et al., 2012) it has been suggested that a switch from interocular to interhemispheric suppression is crucial in the ocular dominance changes induced by MD (Pietrasanta et al., 2014). The neighboring rows of barrels may represent a similar condition for the layer four neurons and therefore we conclude the observed widening of increased activation in response to the spared row stimulation may be accompanied by the interhemispheric inhibition increase, observed only when bilateral stimulation is applied. Additional studies of the electrophysiological properties of the responses are required to determine the nature and location of the inhibitory or excitatory activity involved and the time-scale of the response. Neverthelss, our results support the idea that cortical plasticity is sensitive to bilateral interactions, and is probably the outcome of the counterbalancing effects of interhemispheric activity. Therefore there is nothing like “control hemisphere” since any plastic changes in one hemisphere have to have implications for the functioning of the opposite one. The invovlement of hemispheric interactions in cortical map rearragement has far reaching implications for rehabiliation therapies following unilateral injuries like strokes (Dietrich et al., 1986; Fernández et al., 2003; Hlushchuk and Hari, 2006; Turco et al., 2019).

It is already well known that ipsilateral stimulation in patients after stroke inhibits the injured cortex rehabilitation due to callosal inhibition from the overactivated intact hemisphere (Wang et al., 2012). However, the fact that the contralateral hemisphere participates in intact cortex plasticity in the context of the barrel field is a new phenomenon. On humans so far it was shown that unilateral application of cathodal direct current improved visual acuity in amblyopic patients (Bocci et al., 2018). Probably this effect was caused by cortical plasticity modifying ocular dominance via transcallosal disinhibition. It may be also connected to another similar characteristic of brain functioning - the interhemispheric integration during the cortical map reorganization. If it were confirmed on humans that both hemispheres participate in unilaterally induced plasticity changes and that the interhemispheric interactions are crucial for its arrangement, this would have an important theaupathic implications.

## Data Availability Statement

The raw data supporting the conclusions of this article will be made available by the authors, without undue reservation.

## Ethics Statement

The animal study was reviewed and approved by the local Ethical Committee for Animal Experiments in Warsaw (The I Lokalna Komisja Etyczna ds. Doświadczeń na Zwierzętach w Warszawie) with the agreement number 270/2012; 15/03/2012.

## Author Contributions

JJ: experimental project, 2DG brain mapping and histologial staining performance, data acquisition and analysis, manuscript preparation. RB: optical density measurement analysis. MK: methodological set up of the 2DG brain mapping. MS: histological staining, data acquisition, data analysis and preparation for the manuscript. WS: statistics and data analysis. AS: experimental methodological set up and data acquisition and preparation for the manuscript. PU: data analysis and statistic. All authors contributed to the article and approved the submitted version.

## Conflict of Interest

The authors declare that the research was conducted in the absence of any commercial or financial relationships that could be construed as a potential conflict of interest.
