# Supplementary material for: The Role of Interhemispheric Interactions in Cortical Plasticity
Source: Front Neurosci. 2021 Jul 9;15:631328. doi: 10.3389/fnins.2021.631328 (PMC8299724; doi:10.3389/fnins.2021.631328)
Supplement: Supplementary file 1 [file Data_Sheet_1.pdf]

layers	types of row B whiskers stimulations	row B representation width [µm]		interhemispheric difference of row B representation [%]	significance of the difference
		hemisphere			
		undeprived	deprived		
II/III	bilateral	922 ± 107	1249 ± 230	41 ± 20	p = 0.003
	unilateral	842 ± 65	988 ± 136	13 ± 9	p = 0.04
IV	bilateral	808 ± 42	1176 ± 125	46 ± 19	p = 0.0002
	unilateral	837 ± 54	964 ± 73	19 ± 7	p = 0.001
V/VI	bilateral	839 ± 105	1095 ± 187	30 ± 7	p = 0.0003
	unilateral	749 ± 67	863 ± 78	17 ± 8	p = 0.008

Supp. Tab. 1: Spared and undeprived rows B cortical representations width showing the percentage of interhemispheric difference and its significance; p<0.05

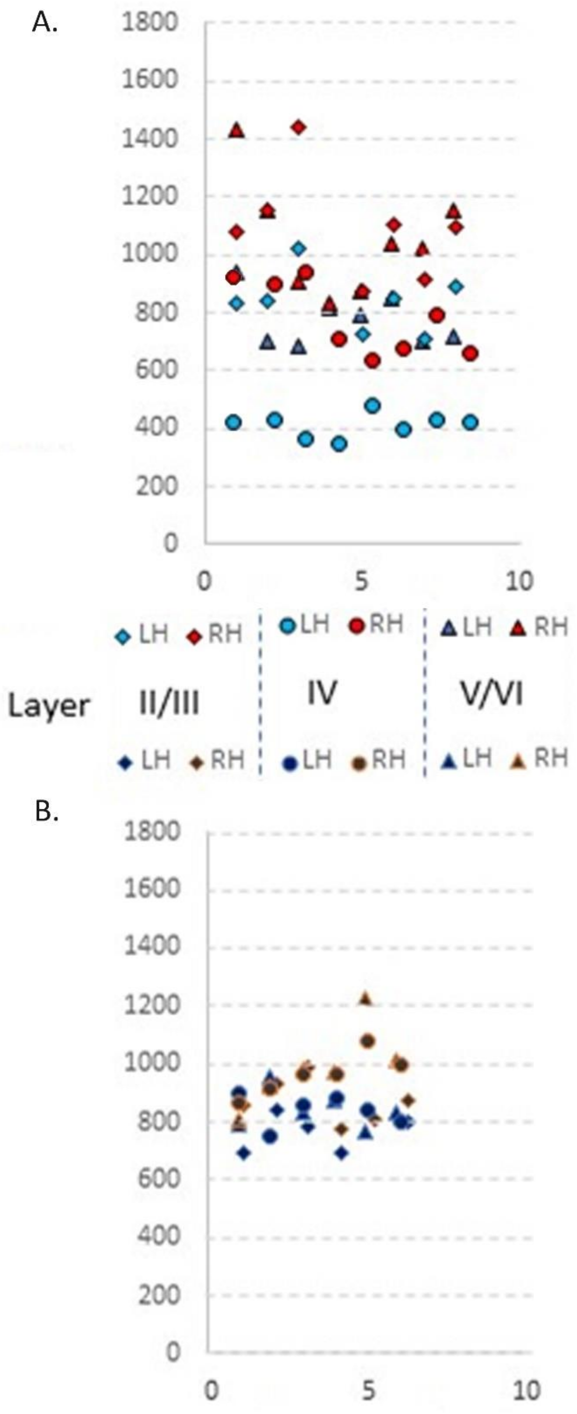

Supp. Fig. 1: Width of 2DG incorporation in row B representations in the contralateral, deprived (red) and non-deprived (blue) hemispheres and its dependence on uni- (A) vs. bilateral (B) whiskers stimulation in layers II/III (squares), IV (circles) and V/VI (triangles). Experimental groups were deprived and bilaterally (n=8) or unilaterally (n=6) stimulated during brain activity mapping; ANOVA with paired T-test and bootstrap analysis were used. * Mean ± SD; * P < 0.05.
